# A dataset of sandfly (Phlebotomus papatasi, Phlebotomus alexandri, and Phlebotomus sergenti) genital and pharyngeal images

**DOI:** 10.1016/j.dib.2024.111031

**Published:** 2024-10-16

**Authors:** Mohammad Fraiwan, Rami Mukbel, Dania Kanaan

**Affiliations:** a Department of Computer Engineering, Jordan University of Science and Technology, P.O. Box 3030 Irbid 22110 Jordan; b College of Veterinary Medicine, Jordan University of Science and Technology, P.O. Box 3030 Irbid 22110 Jordan

**Keywords:** Artificial intelligence, Sexing, Sandfly, Classification, Taxonomy, Entomology

## Abstract

Sandflies serve as carriers for numerous tropical diseases, including leishmaniasis, bartonellosis, and sandfly fever. Furthermore, sandflies are species-specific when it comes to transmitting corresponding pathogen species. Hence, accurate classification and identification of sandfly species and gender are essential for various purposes such as disease monitoring and control, population management, research and development, and epidemiological investigations. Most of the sexing and taxonomy keys are based on internal morphological features, which may lead to errors due to some features being missed by the naked eye. In this paper, we describe the process we used to collect and prepare samples of three sandfly species (*Ph. alexandri, Ph. papatasi*, and *Ph. sergenti*). The dataset described in this article contains two images per sample, representing the pharynx in the head and the genitalia in the abdomen. The dataset is organized into male and female categories for each of the three species. The sex and species were determined manually by two specialists. This dataset can be used to develop automated methods for sex identification and taxonomy. Additionally, it can be used to train students in speciation and taxonomy. To the best of our knowledge, this is the first publicly available dataset of images of this kind.

Specifications Table

**Table:** 

Subject	Computational Biology
Specific subject area	The dataset can fall into areas relating to taxonomy, gender identification, entomology, and artificial intelligence applications
Type of data	Image Raw, Processed
Data collection	An Optika dissecting microscope was used to cut the head and genital segments. The data was acquired by photographing mounted adult sandfly body parts using Motic BA200 compound microscope at appropriate magnification and lighting.
Data source location	1. Institution: Jordan University of Science and Technology City: Irbid Country: Jordan Latitude: 32.493428 and longitude: 35.987968 2. Samples were collected from north Jordan, Irbid Waqqas newline Coordinates in decimal degrees (latitude: 32.5427, longitudinal: 35.60533).
Data accessibility	Repository name: Mendeley Data Data identification number: 10.17632/j9srxj9mvd.2 Direct URL to data: https://data.mendeley.com/datasets/j9srxj9mvd/2 Instructions for accessing these data: None.
Related research article	Journal: N/A DOI: N/A

## 1 Value of the Data

• The dataset is useful for developing automated methods and systems for the taxonomy and sex identification of sandfly species based on genital and pharyngeal images. It serves multiple purposes, including the construction of an expanded dataset encompassing more sandfly species. Furthermore, the images can be used in segmentation and image processing applications. Additionally, it provides a valuable resource for training entomology students in the sexing and speciation of sandflies.
• This dataset will benefit entomologists and artificial intelligence researchers interested in designing automated methods for sandfly species taxonomy and gender identification. This, in turn, will indirectly aid agencies and individuals interested in disease surveillance and control, managing breeding and populations, research, and development. Moreover, biology students and entomology researchers can use the dataset for training in the identification of insects at the genus and species levels.
• The data can be reused by itself to design new artificial intelligence models for the taxonomy and gender identification of sandflies. Moreover, it can be useful to develop segmentation or object detection algorithms to extract relevant and important parts of the images. Furthermore, it can be used to expand existing datasets by adding more images/sandfly species. In addition, the stitched images can be useful in research that employs early fusion of multiple images.
• The data can be reused by itself to design new artificial intelligence models for the taxonomy and gender identification of sandflies. Moreover, it can be useful to develop segmentation or object detection algorithms to extract relevant and important parts of the images. Furthermore, it can be used to expand existing datasets by adding more images/sandfly species. In addition, the stitched images can be useful in research that employs early fusion of multiple images.

## 2 Background

Sandflies are vectors for several tropical diseases, including leishmaniasis, bartonellosis, and sandfly fever. Furthermore, sandflies exhibit species-specificity in transmitting particular pathogens, with females being responsible for disease transmission. Therefore, accurate classification of sandfly species and sex identification are crucial for disease surveillance and control, managing populations, research and development, and conducting epidemiological studies. Currently, this classification is typically performed manually by observing internal morphological features, which can be a tedious and error-prone process. This motivated us to design artificial intelligence-based systems for the automatic sexing and taxonomy of sandflies. To achieve this, a dataset of sandfly morphological features was needed. To the best of our knowledge, there are no publicly available datasets of sandfly pharyngeal and genital images that would facilitate the advancement of AI applications in this field.

## 3 Data Description

The dataset includes genital and pharyngeal images from 758 sandfly insects [1]. Each sandfly contributed one genital and one pharyngeal image. Hence, there was no repetition or multiple images from the same sandfly per body part. The number of images per species and sex is shown in Table 1.

**Table 1:** Details of the species and sex of the sandfly dataset.

Specie	Sex	No. of Images
*Ph. alexandri*	Male	85
*Ph. alexandri*	Female	106
*Ph. papatasi*	Male	158
*Ph. papatasi*	Female	269
*Ph. sergenti*	Male	95
*Ph. sergenti*	Female	45

**Fig. 1:**
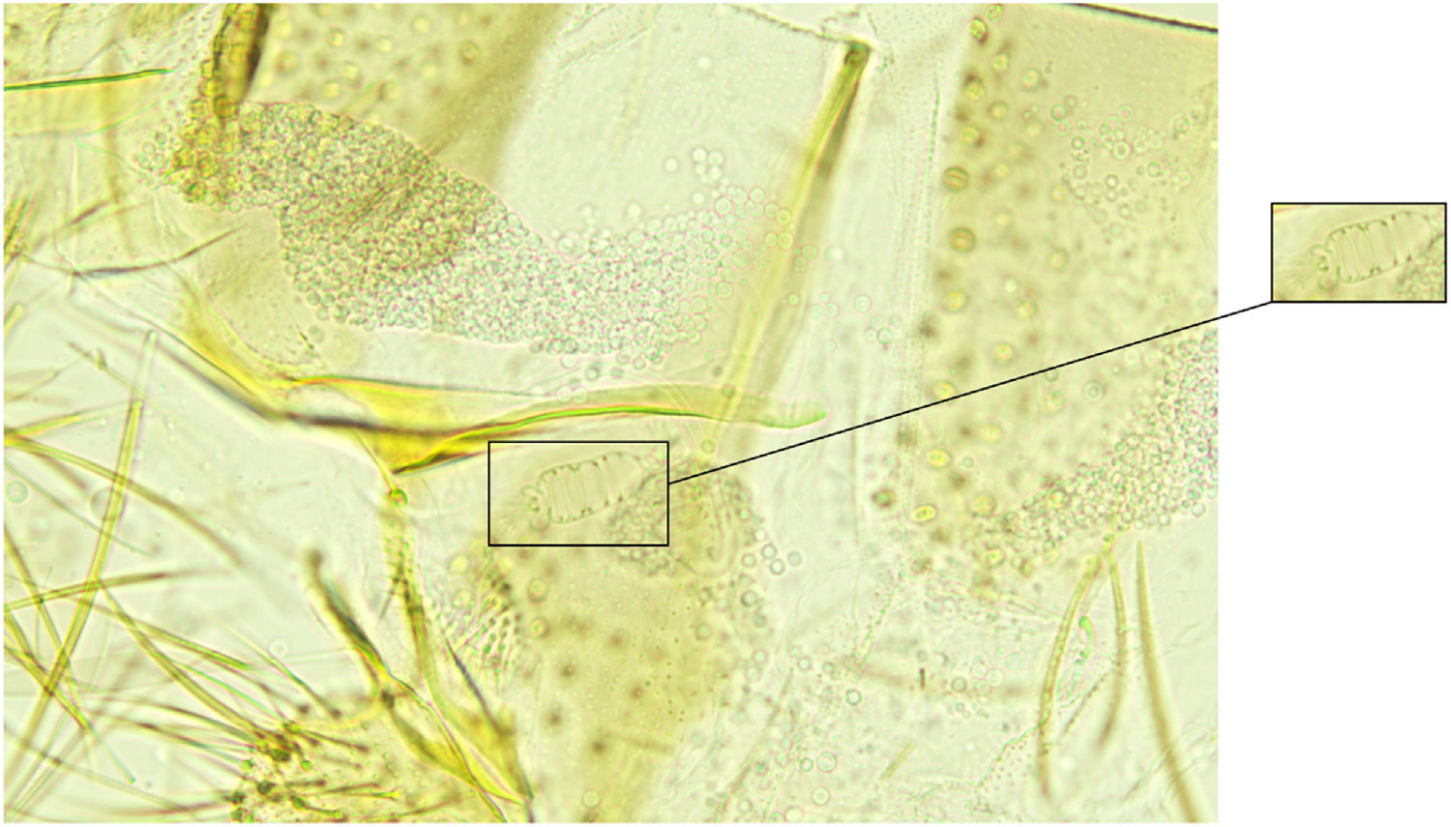
An unedited original image of a *Ph. alexandri* female genitalia, with the region of interest marked and extracted alongside.

**Fig. 2:**
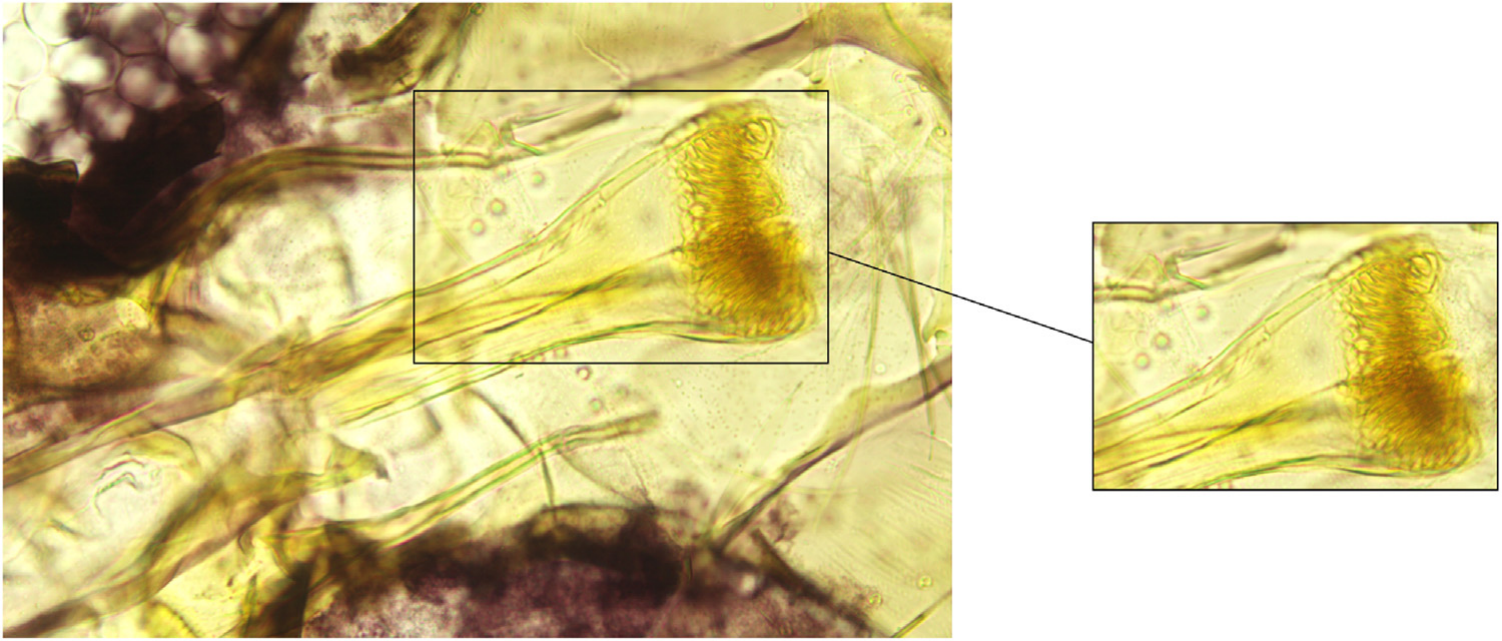
An unedited original image of a Ph. alexandri female pharynx, with the region of interest marked and extracted alongside.

**Fig. 3:**
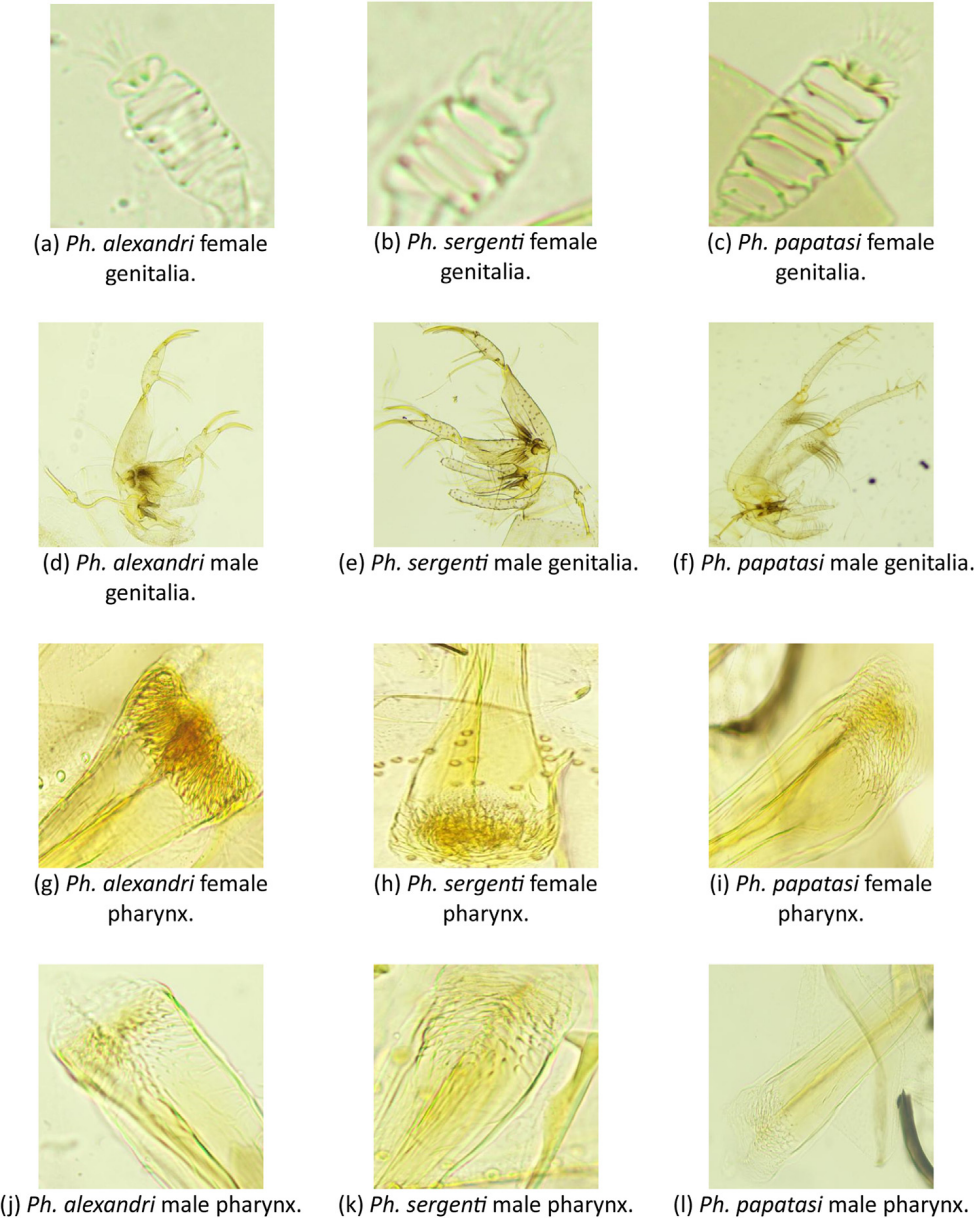
Sample cropped images from the dataset of the pharyngeal and genital regions, representing both sexes across the three species.

**Fig. 4:**
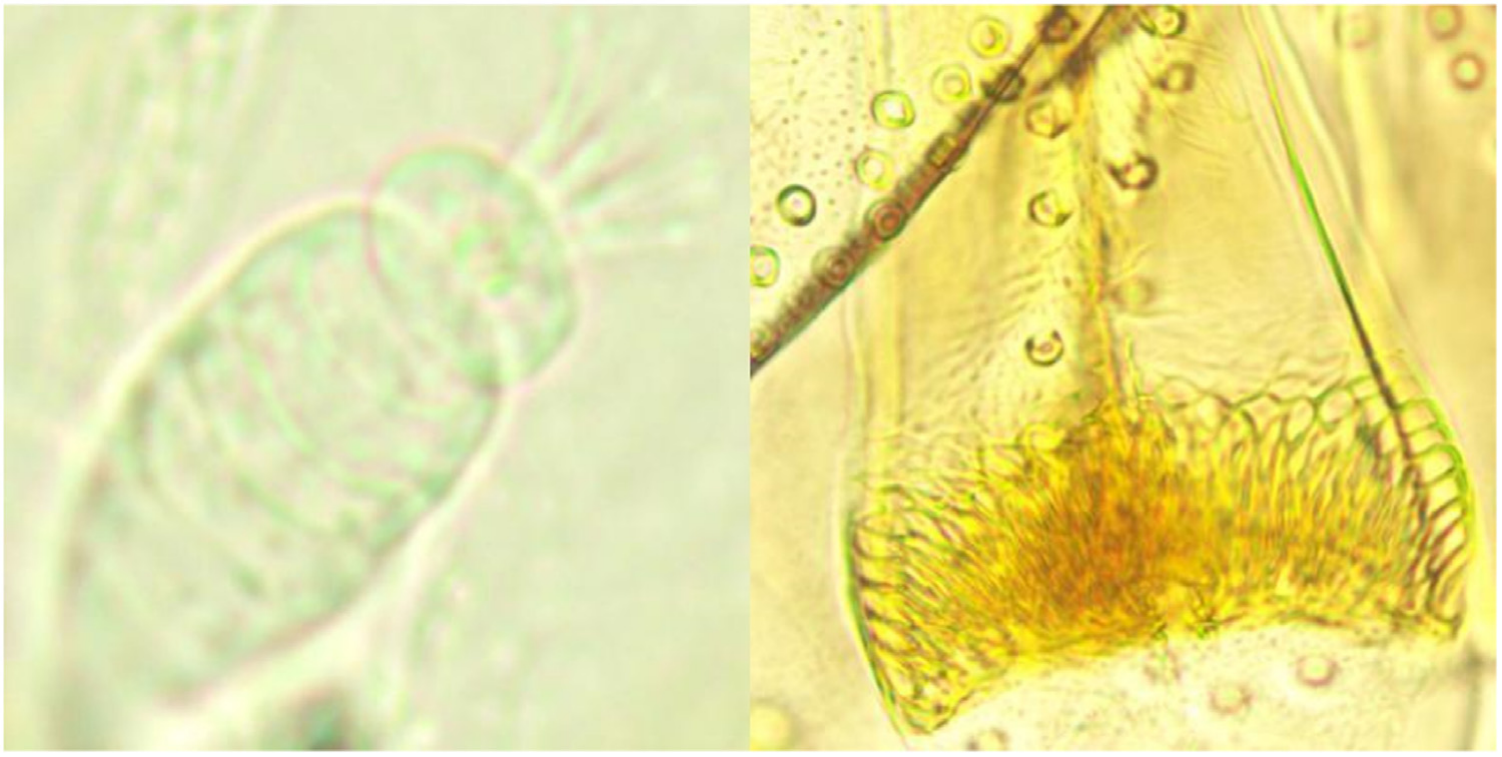
A stitched *Ph. alexandri* female genitalia and pharynx.

The dataset is organized into three separate folders (i.e., ‘Originals’, ‘Cropped’, and ‘Stitched’), each representing a different version of the images in a specific form. The ‘Originals’ and ‘Cropped’ folders are organized in a similar fashion. Each contains two subfolders, one called ‘Genitalia’ and the other ‘Pharynx’. These subfolders are further divided into six subfolders corresponding to the sex and species listed in Table 1. The ‘Originals’ folder contains the original images as taken by the microscope camera with no editing (see Fig. 1, Fig. 2). The ‘Cropped’ folder contains the cropped versions of the images, focusing on the part of interest (i.e., genitalia or pharynx) as much as possible (see Fig. 3). Each cropped image has a dimension of 400 × 400 pixels. The ‘Stitched’ folder contains the cropped images combined (i.e., stitched) so that the genital image and the corresponding pharyngeal image are in one image of 400 pixels in height and 800 pixels in width as an RGB image, see Fig. 4. Hence, the ‘Stitched’ folder contains only six subfolders corresponding to the sex and species listed in Table 1.

Each version of the images serves a specific purpose in research. The original images can be utilized to develop image segmentation methods aimed at automatically extracting (i.e., cropping) relevant regions. This would involve manually annotating the images with bounding boxes, representing the ground truth for training segmentation algorithms. On the other hand, the manually cropped images in the dataset, prepared by the authors, are ready to be used in classification algorithms that identify the sex and species of the sandfly based on the pharynx image, genitalia image, or both. Additionally, identification keys suggest that using both pharyngeal and genital images enhances accuracy in species identification. One potential approach to this is image stitching, which combines the two image types into a single image, beneficial for algorithms that require a single image input rather than multiple inputs.

## 4 Experimental Design, Materials and Methods

**Fig. 5:**
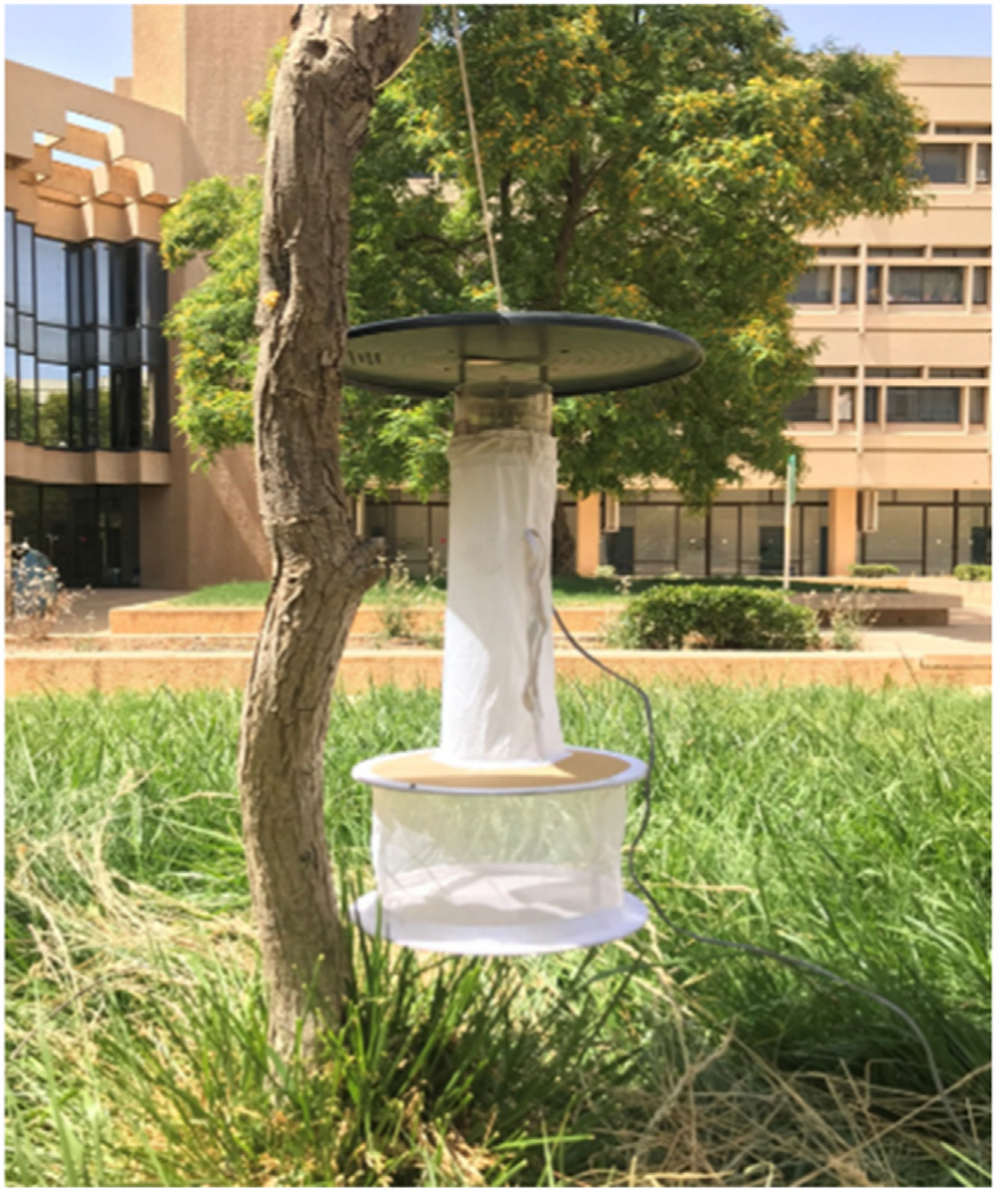
One of the CDC light traps used to catch the sandflies.

Sandfly samples were collected by placing a Centers for Disease Control and Prevention (CDC) light trap in specific locations from sunset to sunrise at an elevation of one meter from the ground (see Fig. 5). Specific environmental conditions need to be met for optimal collection. Understanding these conditions can be crucial for planning successful outings. In this case, hot nights, where the temperature is in the mid to upper twenties Celsius, with calm or no winds, yielded the most catch. Moreover, it is necessary for the trap light to be the only source of light in the vicinity of the trap. The next morning, the traps were collected and transferred to the laboratory for further examination and processing. The traps must be placed in a -20°C freezer for at least half an hour to ensure that the insects are dead before starting the practical work. After half an hour, each trap was emptied onto white paper, and the sandflies were individually transferred into 1.5 mL microcentrifuge tubes. Each tube was then filled with a prepared clearing solution containing chloral hydrate and phenol, which makes the fly bodies transparent to expose the internal morphological features for further identification.

The sandflies remain in the clearing solution for two days. After that the specimens are ready to be dissected. Using an Optika dissecting microscope, fine needles were used to cut the head and the last abdomen segment. These parts are then placed on a clean slide with a drop of purification medium to permanent the specimen. Then, a cover slip is placed on top of the slide, which makes it ready for imaging. Each slide is labelled with a sequential sample number along with the date of trapping. Mounted adult sandflies were scanned using the Motic BA200 compound microscope at different magnifications with sufficient lighting. After that, the samples were identified into their genus-level species and sex using taxonomical keys. The identification of the sandfly species was done based on the following specification for each one:

1. Ph. alexandri [2]
  • Female: The pharynx is about 0.17 mm long, with a straight hind edge and a conical shape. The pharyngeal armature consists of broad scales without a fringe of minute setae, see Fig. 6a. The combined length of A3 + A4 is shorter than the labrum and features short, blunt ascoids. The posterior margin of the pharynx is straight, and the pharynx itself is triangular with straight sides. The mesonotum is dark. The spermatheca has at least four well-defined segments, see Fig. 6b.

**Fig. 6:**
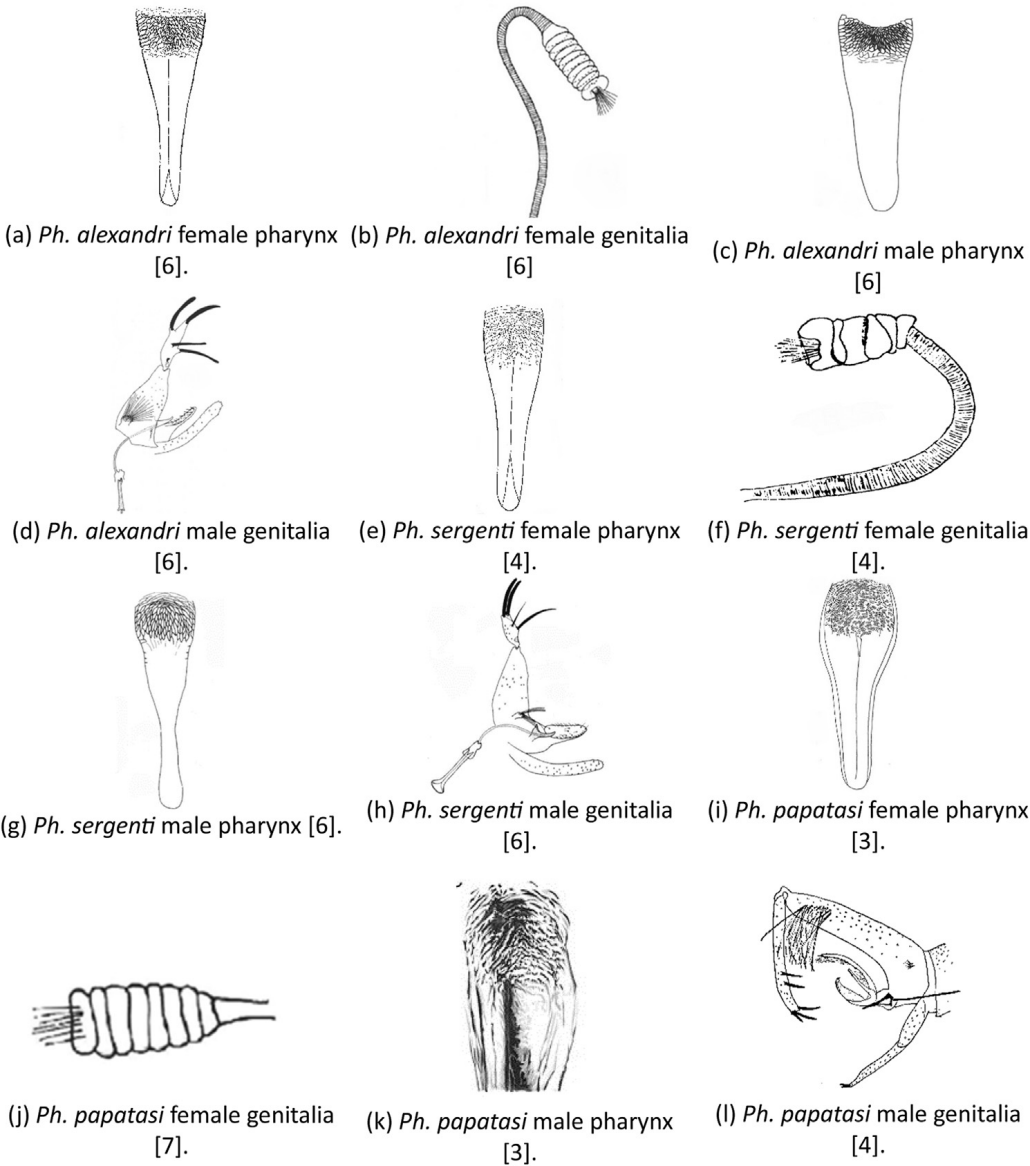
Sandfly identification keys.
  • Male: The gonostyle is broad, with four long, slender spines; the surstyle lacks apical spines, and the paramere is simple. A3 is short and thick, with short, fingerlike ascoids, see Fig. 6c. The gonocoxite tuft is short and broad. The barrel of the sperm pump is not much longer than it is wide, see Fig. 6d. The gonostyle is four times as long as it is thick, with one spine located terminally.
2. Ph. sergenti [3]
  • Female: The pharynx is armed with elongated pharyngeal teeth, see Fig. 6e. The flagellomere 3 is about 0.25 mm long. The genitalia spermathecae have six segments. The pharyngeal armature is about one-fourth the length of the pharynx, with a few large teeth oriented backward. The genitalia spermathecae have about four to five rings, see Fig. 6f.
  • Male: The style appears short with four long spines: two apical and the internal spine is more basal than the external spine, see Fig. 6g. The paramere lacks dorsal ramifications [4]. The lateral lobe lacks short distal spines, see Fig. 6h.
3. Ph. papatasi [5]
  • Female: the pharyngeal teeth in the optical segment appear in horizontal scales or like a network of many lines, with ultimately serrated teeth in a scale shape. pharyngeal teeth are not backward pointed, see Fig. 6i. Genetalia spermathecae: cylindrical shape and contains eight to eleven segments [6], see Fig. 6j. Terminal knob is crowned by a tuft of hair. Their spermathecal ducts are narrow and heavily covered by chitin.
  • Male: Flagellomeres FIII length between 0.27 and 0.34 mm. Wing is about 2.2 and 2.7 mm in length. Eyes look small because the head is relatively long, see Fig. 6k. Genitalia: Second segment of the upper clasper longer than the lower one, see Fig. 6l. Spines are five, three of them are terminals and two short medial [7].

## Limitations

The data collection process and the acquisition of images suffered from several intrinsic problems that reduced the total number of useful images and the clarity of the features of interest, including:

• The body parts in the images (i.e., pharynx and genitalia) are three-dimensional (3D). However, the image capturing was done in two dimensions (2D). Nonetheless, 2D projections may suffice for most applications for this type of image.
• The features of interest in the images maybe jumbled together, which may obscure the identification of morphological features, or may appear distantly separated. Furthermore, the transparent nature of the slides means that some portion may appear under other artefacts.
• The subjects in the slides may fall into different orientations, which may render some slides useless or some images clearer than others. In addition, this will affect the uniformity of the images.
• Due to the dissection process and the other steps in preparation of the slides, many artefacts appear in the images, see Fig. 1, Fig. 2.

## Ethics Statement

All authors have read and followed the ethical requirements for publication in Data in Brief and confirming that the current work does not involve human subjects, animal experiments, or any data collected from social media platforms.

## CRediT Author Statement

Mohammad Fraiwan: Conceptualization, Funding acquisition, Project administration, Methodology, Visualization, Software, Data curation, Writing - Original draft preparation. Rami Mukbel: Conceptualization, Project administration, Writing - Reviewing and Editing, Resources, Validation. Dania Kanaan: Investigation, Data curation, Writing - Original draft preparation, Writing Reviewing and Editing.

## Data Availability

Mendeley DataA dataset of sandfly (Ph. papatasi, Ph. alexandri, and Ph. sergenti) genital and pharyngeal images (Original data)
